# Changes in gene expression and apoptotic response in *Spodoptera exigua* larvae exposed to sublethal concentrations of Vip3 insecticidal proteins

**DOI:** 10.1038/s41598-017-16406-1

**Published:** 2017-11-24

**Authors:** Patricia Hernández-Martínez, Joaquín Gomis-Cebolla, Juan Ferré, Baltasar Escriche

**Affiliations:** ERI de Biotecnología y Biomedicina (BIOTECMED), Department of Genetics, Universitat de València. Dr Moliner, 50, Burjassot, 46100 Spain

## Abstract

The insecticidal Vip3 proteins from *Bacillus thuringiensis* (Bt), along with the classical Bt Cry proteins, are currently used in Bt-crops to control insect pests, since they do not share the same mode of action. Here we characterized the response of *Spodoptera exigua* larvae after Vip3 challenge. The expression profile of 47 genes was analyzed in larvae challenged with three concentrations of Vip3Ca. Results showed that the up-regulated genes were mainly involved in immune response, whereas the down-regulated genes were mainly involved in the digestion process. Other mechanisms of cellular response to the damage such as apoptosis were analyzed. For this analysis, sections from the midguts were examined by terminal deoxynucleotidyl transferase dUTP nick end labeling (TUNEL) staining. The nuclei of the midgut epithelial cells were stained at the highest concentration of the Vip3Ca protein and at lower concentrations of Vip3Aa in agreement with the different potency of the two proteins. In addition, apoptosis was also examined by the analysis of the expression of five *caspase* genes. The present study shows that exposure of *S. exigua* larvae to sublethal concentrations of Vip3 proteins activates different insect response pathways which trigger the regulation of some genes, APN shedding, and apoptotic cell death.

## Introduction

The concern over the excessive use of chemical insecticides has increased in recent years, due to the ecological impact, as well as to the selection for resistance in field insect populations^1^. Among the different biological alternatives for pest control, one of the most popular is the use of bioinsecticies based on *Bacillus thuringiensis* ^2^. This bacterium produces a wide range of insecticidal proteins which are active against a number of agricultural pest species^3^. Insecticidal crystal proteins (Cry proteins) are being used to control insect pests in formulated sprays since 1938^4^ and, since 1996, they have been expressed in transgenic crops to protect them from insect attack^5^. More recently, a novel class of insecticidal proteins (Vip3 proteins) have also been introduced in transgenic crops to complement the toxic action of the Cry proteins, as well as to reduce the risk of insect resistance development in the field^6^ (ISAAA GM Approval Database. http://www.isaaa.org/gmapprovaldatabase).

Cry proteins are highly specific against their target insects and are generally recognized as pore-forming toxins (PFTs)^7^. The mode of action of these proteins has been extensively studied for more than 20 years, especially for Cry1A proteins^8,9^. In general, it is accepted that these toxins are solubilized in the insect gut and then activated by the action of digestive enzymes. The active forms bind to specific receptors in the brush border of epithelial midgut cells and induce pores in the membrane which eventually lead to septicemia and insect death^9^. Nowadays, different models have been proposed to explain how these proteins exert their cytotoxicity, however some aspects remain unclear^8,10^. Much less is known about the mode of action of Vip3 proteins. The Vip3Aa protein was the first member of the family of Vip3 proteins being described^11^ and most studies dealing with the mode of action of Vip3 proteins have been performed with this protein^12^. The available information supports that these proteins act by forming pores in the midgut epithelial cells^13,14^. As for Cry proteins, Vip3 proteins are synthesized as protoxins which are processed by proteases in the larva midgut rendering the active form, which then binds to specific receptors in the brush border of epithelial midgut cells^15–18^. After binding, the Vip3 protein induces disruption of the midgut epithelial cells^18–21^ by its pore forming activity^13,14^. One very interesting feature of the Vip3 proteins mode of action is that they do not share membrane binding sites with Cry proteins, a property which does not only complement the spectrum of activity of Cry proteins, but also decreases the chances of cross-resistance^13,14,17,22,23^.

The insect gut is not only an organ of digestion but also constitutes the first physical barrier that protects the host against penetration of both pathogenic and commensal microorganisms^24,25^. In mammals, some studies have identified the mechanisms that regulate gut mucosal immunity, revealing a central role of innate immunity in these processes^26^. Despite the fact only few studies have been conducted in insects, the available data suggest that midgut epithelial tissue of the insects challenged with either pathogenic or nonpathogenic bacteria^27–29^ is able to trigger an immune response to reduce the cellular and tissue damage. Therefore, the insecticidal activity of Cry and Vip proteins might be affected by the host defense response, since they exert their toxic action in the midgut of the target insects.

Transcriptomic and proteomic approaches are being helpful to elucidate which mechanisms are involved in the host responses to *B. thuringiensis* proteins in non-model insects of agricultural importance^30–37^. In general, these analyses point out that, after protein exposure, the insects usually increase their immune function in addition to reduce their digestive activity^38,39^. Some reports have shown that the expression of some components of the mitogen-activated protein kinase (MAPK) cascade were up-regulated in response to Cry proteins in Coleoptera, Diptera, and Lepidoptera^40–43^. Additionally, other gene families have been described to be transcriptionally regulated in response to Cry and Vip3 proteins in *Spodoptera exigua* larvae^30,31,44–46^, such as the *response to pathogens* (*REPAT*) genes, though their specific role in host response still remains unclear^38,45^.

Apoptosis has also been described as a mechanism of cellular response after the exposure of cultured cells with different PFTs^47–50^. *In vitro* experiments with midgut primary culture cells from *Heliothis virescens* showed that Cry toxins induced apoptosis in epithelial cells^51^. More recently, similar results were observed when CF1 or Sf9 cultured cells were exposed to Cry1A or Vip3Aa proteins, respectively^52,53^. *In vivo* assays also showed that apoptosis could be observed in insect midgut epithelial cells when Cry proteins were administered to both *Culex pipiens* and *Bombyx mori* larvae^54,55^.

The activation of different mechanisms of response in *S. exigua* larvae after the exposure to different *B. thuringiensis* proteins (Cry and Vip3 proteins) has been reported^30,31,38,56^. These mechanisms of response might contribute to reduce the damage produced by *B. thuringiensis* proteins to the insect. To date, the genes identified in the *S. exigua* response to *B. thuringiensis* proteins have been found to be involved in many different aspects of the insect biology such as: metabolism, immune-response, detoxification, and epithelial renewal, among others^38^. The present work extends previous studies carried out on *S. exigua* larvae with the analysis of the expression profile of 47 genes after Vip3Ca exposure. These selected genes were previously found differentially expressed after the exposure to other *B. thuringiensis* proteins and other pathogens^31,56,57^. Thus, the data obtained in the present study could help understand whether the *S. exigua* response to Vip3Ca is specific or, on the contrary, it is a conserved feature independent of the toxic agent to which the insects are exposed to. Furthermore, the damage produced by Vip3Ca and Vip3Aa proteins has been characterized by measuring the *in vivo* response of the midgut epithelial cells (APN shedding and apoptosis).

## Results

### Growth inhibition assays

**Figure 1:**
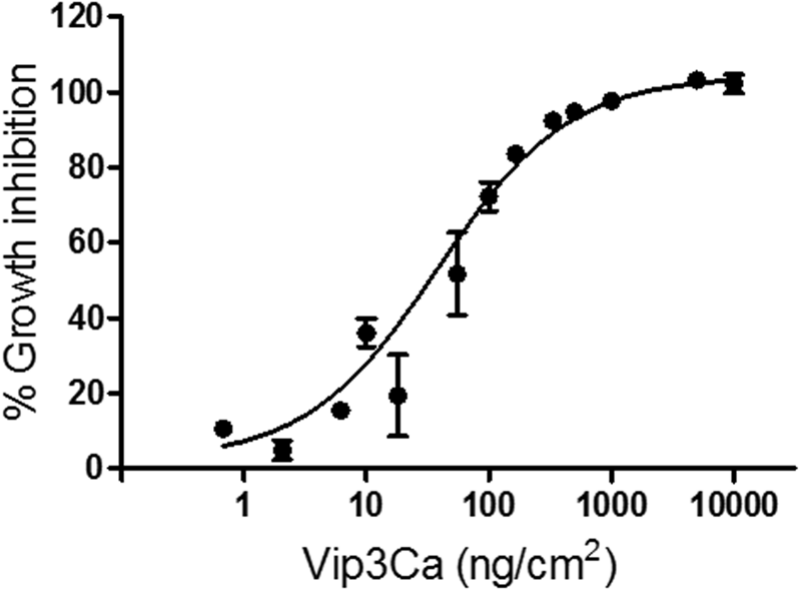
Growth inhibition dose-response curve of newly molted 4th instar *S. exigua* larvae after exposure to Vip3Ca for 24 h. Each value represents the mean of at least three independent experiments (±SEM).

Susceptibility of *S. exigua* 4^th^ instar larvae against the Vip3Ca protein was determined in terms of the effect on larval growth inhibition. The results showed a dose-response relationship, with an EC_50_ of 38.4 ng/cm^2^ (Fig. 1). Therefore, despite the fact that Vip3Ca has negligible activity, in terms of mortality, against *S. exigua* ^58^, it has a clear effect on larval growth inhibition.

### Analysis of the gene expression upon Vip3Ca challenge

The expression profile of 47 genes was analyzed in *S. exigua* midguts by qRT-PCR, after 24 h exposure to Vip3Ca. To be able to compare gene expression results with those previously reported after Vip3Aa challenge^31,56^, the concentration of Vip3Ca to cause 99% growth inhibition on 4^th^ instar *S. exigua* larvae (1000 ng/cm^2^) was used. In order to test whether a lower or higher concentration of Vip3Ca could alter the regulatory effect on these genes, their expression levels were also determined after a challenge with either 100 or 10000 ng/cm^2^ of Vip3Ca.

**Figure 2:**
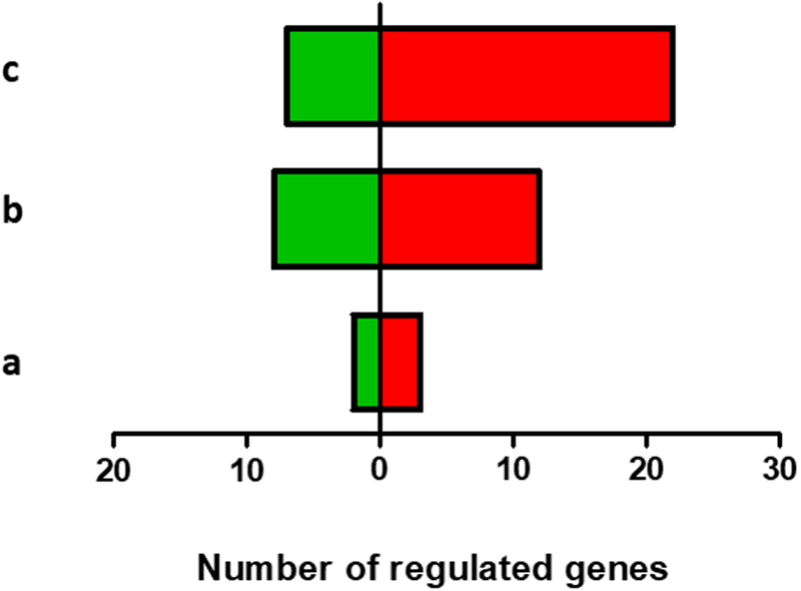
Overview of the regulated genes in *S. exigua* midgut after Vip3Ca challenged at 100 (**a**), 1000 (**b**), and 10000 (**c**) ng/cm^2^. A total of 47 genes were analyzed. The number of up- and down-regulated genes are indicated in red and green bars, respectively.

**Figure 3:**
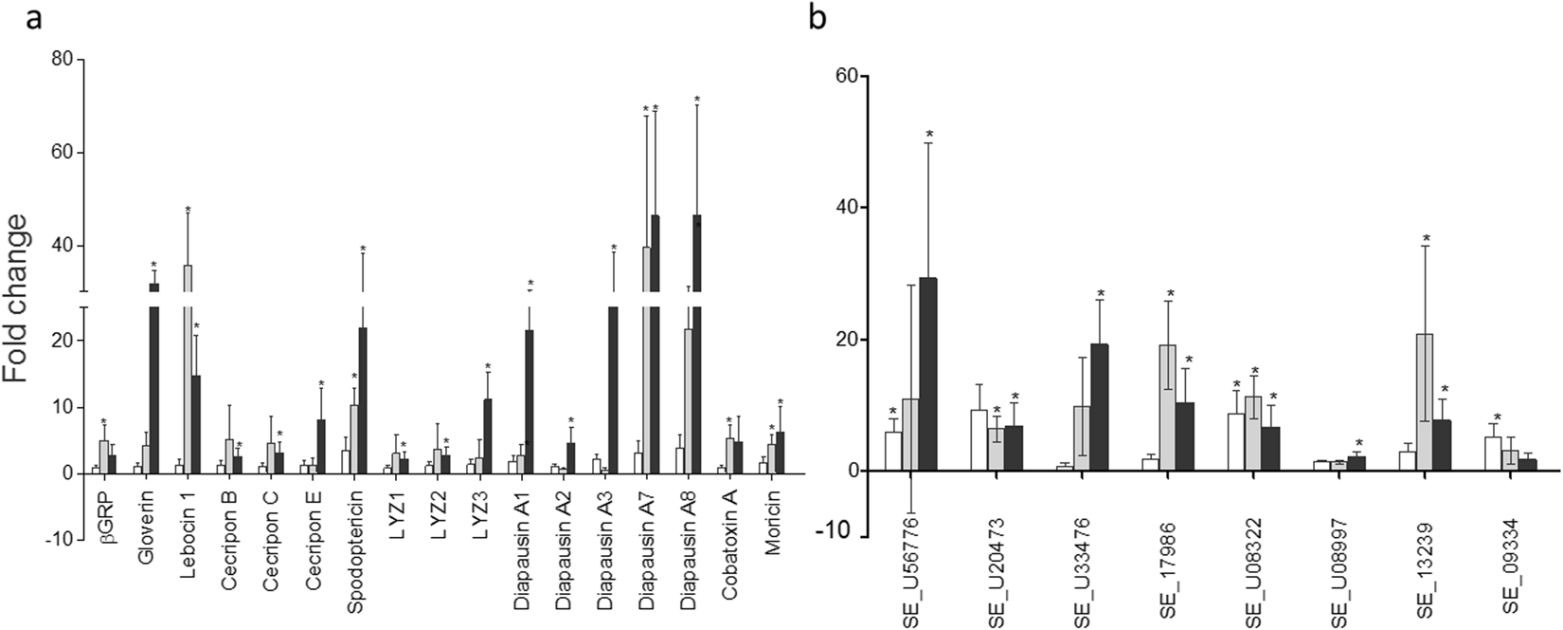
Analysis of the genes whose expression is up-regulated after 24 h exposure at three concentrations of Vip3Ca. (**a**) Immuno-related genes (**b**) Non immuno-related genes. White, grey, and black bars represent the gene expression of each transcript after Vip3Ca challenged at 100, 1000, and 10000 ng/cm^2^, respectively. Abbreviations: βGRP: beta-1,3-glucan recognition protein. The expression of each gene in the gut of Vip3Ca exposed larvae was compared to its control in the gut of control larvae (exposed to WK6). Fold-changes were determined by using the REST MCS software. Each bar represents the mean of three independent experiments (±SD). Significant differences were indicated by an asterisk.

**Figure 4:**
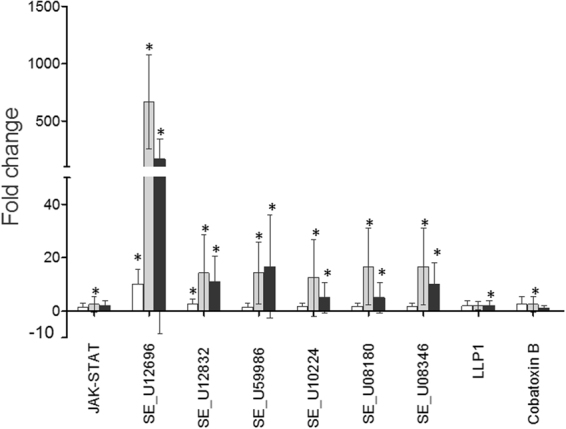
Analysis of the genes whose expression is down-regulated after exposure 24 h at three concentrations of Vip3Ca. White, grey, and black bars represent the gene expression of each transcript after Vip3Ca challenged at 100, 1000, and 10000 ng/cm^2^, respectively. The expression of each gene in the gut of Vip3Ca exposed larvae was compared to its control in the gut of control larvae (exposed to WK6). Fold-changes were determined by using the REST MCS software. Each bar represents the mean of three independent experiments (±SD). Significant differences were indicated by an asterisk.

The results showed that the number of genes whose expression was altered was different depending on the exposure condition (concentration of Vip3Ca) (Fig. 2). At the lowest concentration of Vip3Ca (100 ng/cm^2^), only 5 genes (about 11%) were regulated (Fig. 2 and Supplementary Table S1), whereas at the other two concentrations tested, 1000 and 10000 ng/cm^2^, the number of regulated genes was 20 (about 43%) and 29 (around 62%), respectively. The distribution of up- and down-regulated genes, according to the concentration of Vip3Ca used in each treatment, is summarized in Supplementary Fig. S1. Almost all the regulated genes at 100 and 1000 ng/cm^2^ were also found regulated at 10000 ng/cm^2^ of Vip3Ca, suggesting that the response can be dose-dependent. The ratio of up-regulated and down-regulated genes at 100 and 1000 ng/cm^2^ was similar (3 up- and 2 down-regulated, and 12 up- and 8 down-regulated, respectively). However, at 10000 ng/cm^2^, the ratio of up-regulated genes and down-regulated genes was higher (22 vs. 7, respectively). The levels of transcriptional activation ranged from 2.8-fold to 46.5-fold, whereas the levels of transcriptional repression ranged from 2.5-fold to 653-fold (Figs 3, 4 and Supplementary Table S1).

At 100 ng/cm^2^, the up-regulated genes included a member of the *REPAT* family (*REPAT2*), a gene coding for a juvenile hormone binding protein (SE_U56776), and one for for a pancreatic lipase (SE_U08322). Of the two down-regulated genes, one had homology with a lipase gene, whereas the other two had no homology to known genes from public sequence databases (SE_U12696).

At the concentration of Vip3Ca to provoke 99% growth inhibition on 4^th^ instar *S. exigua* larvae (1000 ng/cm^2^) most of the up-regulated genes (7 out of 12) encoded antimicrobial peptides (AMPs), being the most up-regulated ones *Diapausin A6* and *Lebocin 1*. The gene coding for the beta-1,3-glucan recognition protein (β-GRP) was only slightly overexpressed. Additionally, the other up-regulated genes showed homology with genes that encoded juvenile hormone binding proteins (SE_U17986 and SE_U13239) and for pancreatic lipases (SE_U20473 and SE_U08322). Around 88% of the down-regulated genes showed homology to known genes from public sequence databases, including those coding for lipases, proteases and chitin deacetylases (Supplementary Table S1). The biggest repression (653-fold) was observed for one gene which no homology to known gene (SE_U12696). The *Cobatoxin B* gene, as well as the immune signaling pathway *JAK-STAT* gene, were also found slightly down-regulated.

At the highest concentration used (10000 ng/cm^2^) the scenario observed was similar to the one described for the larvae exposed to 1000 ng/cm^2^. Fifteen of the twenty-two up-regulated genes encoded AMPs (Fig. 3). These 15 AMPs belong to different groups such as the cysteine-rich peptides (Diapausin A1-A3, A6 and A7, and Spodoptericin), glycine- and proline-rich peptides (Gloverin), amphipatic peptides (Cecropin B, C, and E, and Moricin) and lysozymes (LYZ1, 2, and 3). Other up-regulated genes showed homology with genes that encoded juvenile hormone binding proteins (SE_56776, SE_U17986, and SE_U13239), for a Diapausin precursor (SE_U33476), for pancreatic lipases (SE_U20473 and SE_U08322), and for a gene with no homology to any known gene (SE_U08997). The down-regulated genes showed homology with those coding for lipases, proteases and chitin deacetylases (Supplementary Table S1). Again, the biggest repression (167-fold) was observed for the gene SE_U12696. The gene encoding for the AMP LLP1 was also found slightly down-regulated.

### Determination of epithelial damage by APN shedding assays

**Figure 5:**
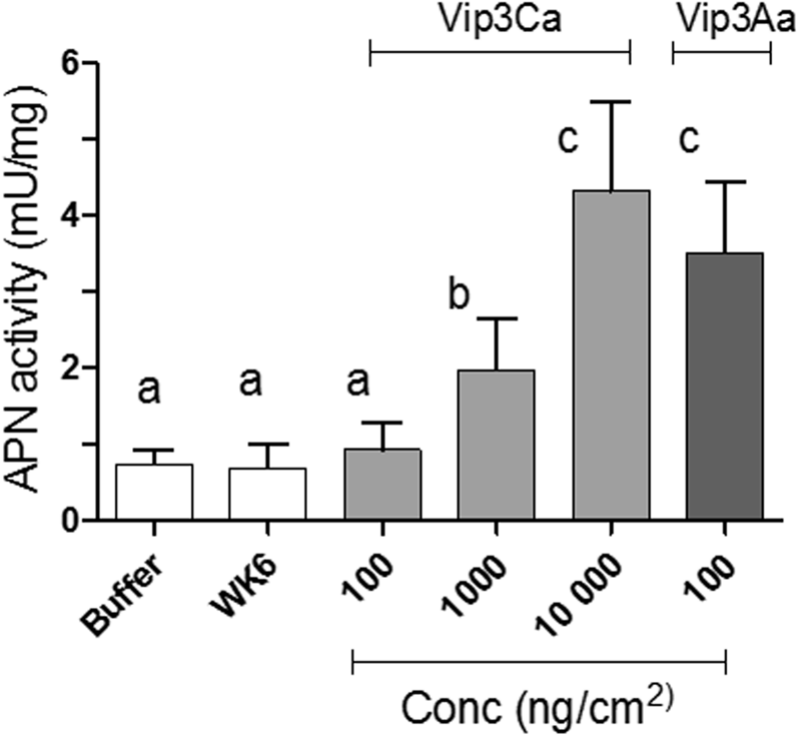
Activity of the APN protein in the midgut lumen of larvae exposed for 24 h to WK6 proteins (control larvae), Vip3Ca (three concentrations) or Vip3Aa (one concentration). Means were compared by one-way ANOVA, followed by Dunnett’s comparison test (*P* < 0.001). Significant differences between control larvae and treated larvae were indicated by different letters.

Shedding of membrane-bound APN to the lumen, as a marker for epithelial damage, was measured after 24 h exposure to Vip3 proteins at the concentration of 100 ng/cm^2^ of Vip3Aa and at 3 different concentrations of Vip3Ca (100, 1000, and 10000 ng/cm^2^). The results showed that, in larvae exposed to Vip3Ca, the APN activity in the luminal fluid increased *ca*. 3-fold and 6-fold at 1000 or 10000 ng/cm^2^, respectively, though no significant change was observed at 100 ng/cm^2^ (Fig. 5). A correlation between growth inhibition and the amount of APN released into the luminal fluid produced by Vip3Ca protein was observed (Supplementary Fig. S2).

The luminal APN activity of those larvae exposed to 100 ng/cm^2^ of Vip3Aa increased *ca*. 5-fold. Interestingly, the APN activity in the lumen fluid of larvae exposed to a concentration that produces a 99% of growth inhibition for each respective Vip3 protein (100 ng/cm^2^ for Vip3Aa and 1000 ng/cm^2^ for Vip3Ca) was significantly higher for Vip3Aa.

### Determination of epithelial cell damage by the TUNEL assay

**Figure 6:**
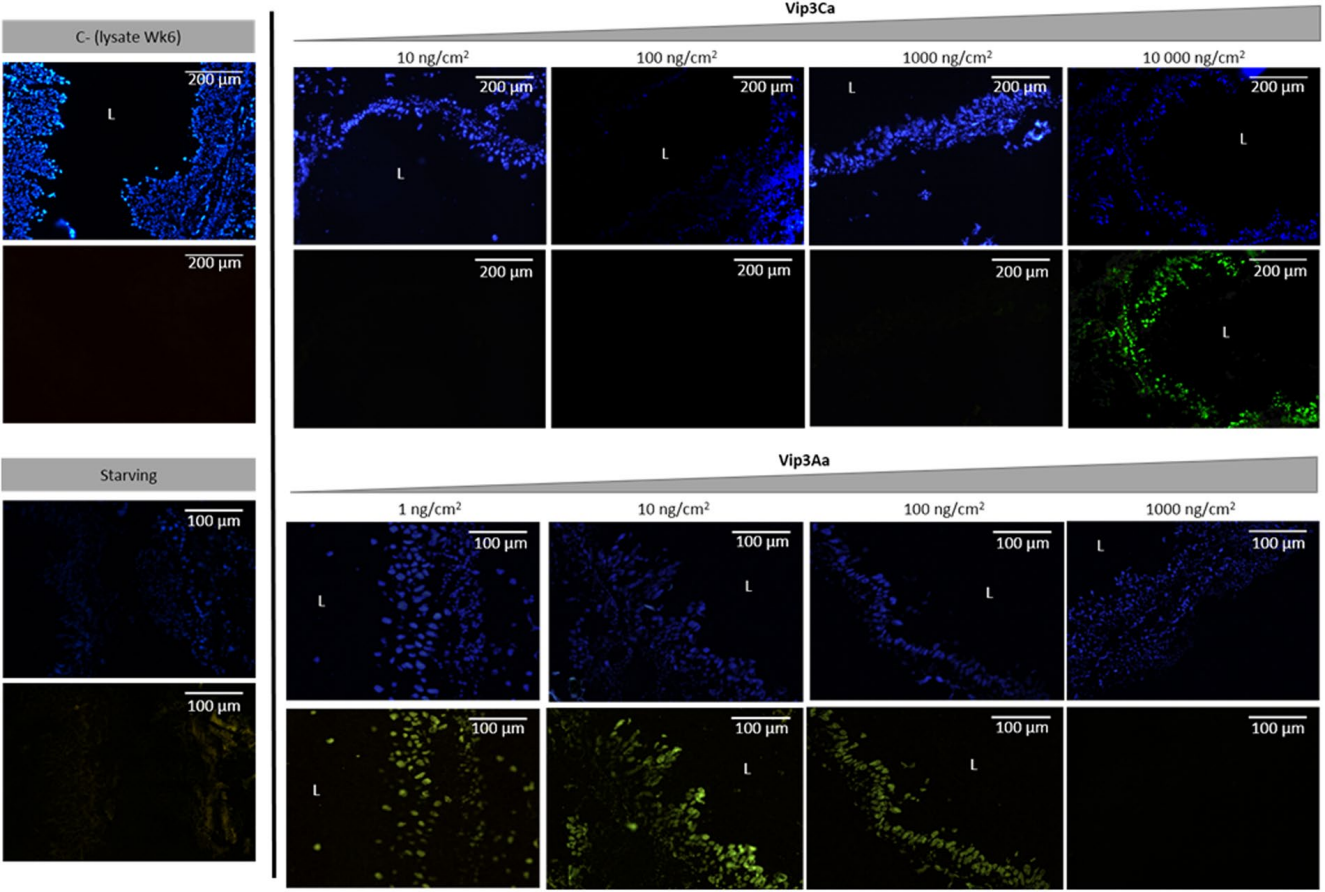
Effect of sublethal concentrations of Vip3Ca and Vip3Aa proteins to *Spodoptera exigua* midgut epithelial cells. L4 newly molted larvae were intoxicated with Vip3 proteins for 24 h and then midgut tissue sections were prepared and stained with TUNEL (green signal) and DAPI (blue signal). As controls, larvae fed with the empty vector (WK6) and 24 h starving larvae were used. Magnification was 100× for the Vip3Ca protein and 200× for the Vip3Aa protein. L, gut lumen.

To test whether exposure to sublethal concentrations of Vip3Ca could trigger a signaling pathway leading to the death of the epithelial cells by apoptosis, midguts of larvae exposed for 24 h to Vip3Ca were sectioned and stained with the DeadEnd^TM^ Fluorimentric TUNEL system. Midguts of larvae exposed to Vip3Aa were used for comparative purposes. The results showed that in control larvae (exposed to WK6 proteins) and in starving larvae, no TUNEL-positive cells were observed. No TUNEL-positive cells were observed either after Vip3Ca treatment at the two lowest concentrations used (10 and 100 ng/cm^2^). However, a few TUNEL-positive cells were observed in the gut of larvae intoxicated with 1000 ng/cm^2^ of Vip3Ca and, at the highest concentration used (10000 ng/cm^2^), almost all the cells were TUNEL-positive (Fig. 6). In the case of Vip3Aa challenge, TUNEL-positive cells were observed in the gut of the larvae intoxicated with the three lowest concentrations (1, 10 and 100 ng/cm^2^) and no TUNEL-positive cells were found at the highest concentration (1000 ng/cm^2^) (Fig. 6).

### Analysis of the expression levels of apoptosis-related genes in *S. exigua* larval midgut challenged with Vip3Ca

**Figure 7:**
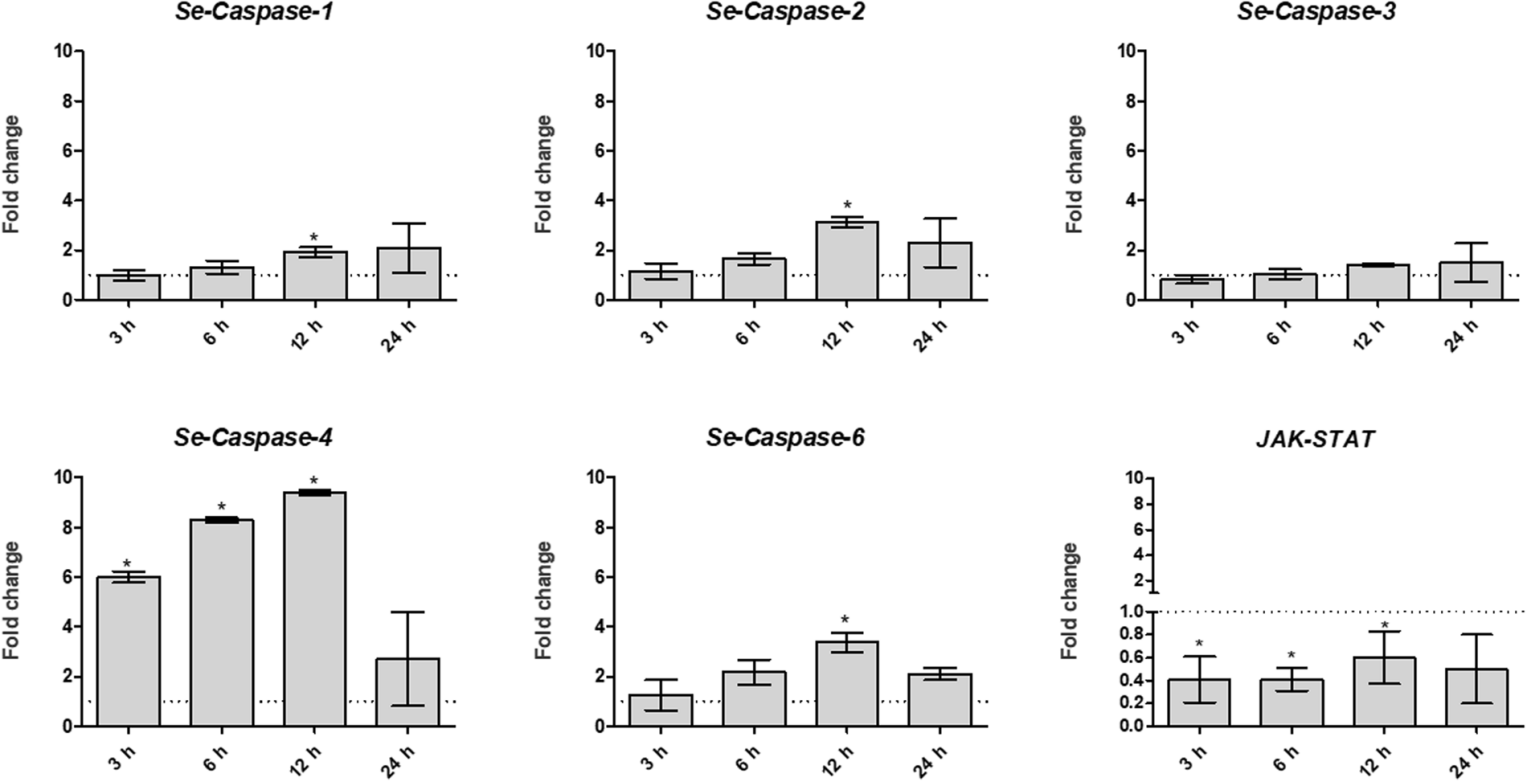
Analysis of the transcriptional induction of apoptotic related genes at 10000 ng/cm^2^ of Vip3Ca for 3 h, 6 h, 12 h, and 24 h. The expression of each gene in the gut of larvae exposed to Vip3Ca was compared to its control in the gut of control larvae (exposed to WK6 empty vector). Fold-changes were determined by using the REST MCS software. Each bar represents the mean of three independent experiments (±SD). Significant differences were indicated by an asterisk.

The induction of the apoptotic process was analyzed in the midgut epithelial cells, at the molecular level, by measuring the change in transcription levels of 5 *caspase* genes by qRT-PCR. The transcription levels of the gene encoding a component of the JAK-STAT pathway was also analyzed since this pathway has been related with the renewal of the midgut tissue^59,60^. To analyze the time course of apoptosis, the expression level of the six genes were monitored at 4 different time points: 3 h, 6 h, 12 h, and 24 h after Vip3Ca challenge at 10000 ng/cm^2^. After either 3 h or 6 h of exposure, only *Se-Caspase-4* was found up-regulated, whereas after 12 h of exposure four of the 5 *caspase* genes studied were found up-regulated (Fig. 7). In contrast, none of the 5 *caspase* genes studied were found regulated after 24 h exposure. These data suggest that the main transcriptional induction of apoptotic machinery occurs after 12 h exposure. The gene coding a component of the JAK-STAT pathway was found down-regulated after 3 h, 6 h, and 12 h of Vip3Ca challenge (Fig. 7).

### Fitness cost analysis

**Figure 8:**
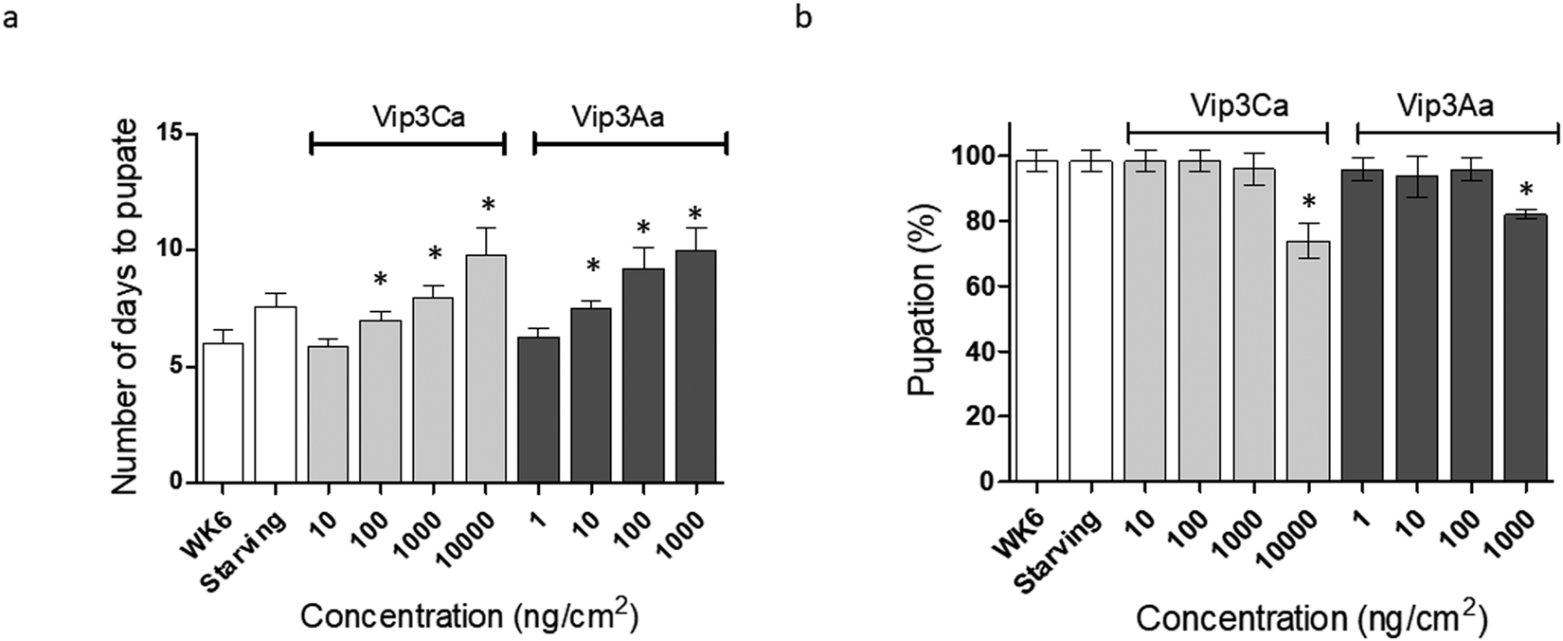
Analysis of the associated fitness cost on *S. exigua* 4^th^ instar larvae after Vip3Ca or Vip3Aa exposure. Two parameters were measured: (**a**) number of days to pupation, and (**b**) % of pupation. Means were compared by one-way ANOVA, followed by Dunnett’s comparison test (*P* < 0.001). Significant differences between control larvae and treated larvae were indicated by an asterisk.

Since the exposure to Vip3 proteins affected the transcriptional pattern of *S. exigua* larvae and caused epithelial and cellular damage, we wanted to determine whether the exposure also had an associated fitness cost. The results showed significant differences in the time to pupation for those larvae exposed to higher concentrations of either Vip3Aa (10, 100, and 10000 ng/cm^2^) or Vip3Ca (100, 1000, and 10000 ng/cm^2^), as compared to control larvae and with larvae exposed to lower concentrations (Fig. 8a). Interestingly, the time to pupation was also found significantly longer in starving larvae. The percentage of pupation was found significantly lower in those larvae exposed to the highest concentration of either Vip3Aa or Vip3Ca (Fig. 8b).

## Discussion

A better knowledge of the different mechanisms of insect response after the exposure to different *B. thuringiensis* proteins will broaden our understanding of how larvae response might help to reduce the damage produced by these insecticidal proteins. In the present study, the expression profile of 47 selected genes was analyzed in *S. exigua* larvae challenged with three concentrations of Vip3Ca. These genes were previously found differentially expressed after the exposure to Vip3Aa, Cry1Ca, and other pathogens^31,56,57^. In order to compare our results with those obtained previously by Bel *et al*.^31^ and by Crava *et al*.^56^, a concentration of Vip3Ca which caused a 99% of growth inhibition was used as a starting point. Thus, the data obtained could help understand whether the *S. exigua* response to Vip3Ca is specific or, on the contrary, it is a conserved feature independent of the toxic agent to which they are exposed to.

The gene expression results showed that at the lowest concentration of Vip3Ca tested (100 ng/cm^2^) the number of regulated genes was lower (about 11%) than when larvae were exposed to 1000 or 10000 ng/cm^2^ of Vip3Ca (around 43% and 62%, respectively). Moreover, almost all the regulated genes at 100 and 1000 ng/cm^2^ were also found regulated at 10000 ng/cm^2^ of Vip3Ca, suggesting that the host response is dose-dependent. In general, our results agree with previous gene expression studies which showed the up-regulation of genes involved in immune system and hormone modulation (e.g. JH binding protein), and the down-regulation of genes involved in the digestion process (e.g. serine proteases) and peritrophic membrane permeability (e.g. chitin deacetylases), upon exposure to different *B. thuringiensis* proteins^28,30,31,35,36,61–65^. The highest value of down-regulation (653-fold) found in this study was obtained for one gene with unknown function when larvae were exposed to 1000 ng/cm^2^ of Vip3Ca. Similar results were observed by Bel *et al*.^31^ when *S. exigua* larvae were exposed to Vip3Aa. This gene encodes a putative protein called REVIP because it was detected in Response-to-Vip intoxication.

In our study we have included different *S. exigua* immune-related genes which were classified in three categories by Pascual *et al*.^66^: (a) pathogen recognition, (b) immune signaling pathways and melanization process, and (c) antimicrobial effectors (Supplementary Table S1). A general upregulation of the immune-related genes was observed after Vip3Ca exposure at 1000 and 10000 ng/cm^2^, though it is worth to note that the genes belonging to the antimicrobial effectors were more clearly regulated after Vip3Ca exposure than the other genes. These antimicrobial effectors are produced to act as barriers against the progress of bacterial infections^67^. Here we analyzed the transcriptional response of 24 genes coding for 21 AMPs and 3 lysozymes. These genes were described in a detailed study performed by Crava *et al*.^56^ where most of the 24 genes were found up-regulated after Vip3Aa exposure at a concentration to provoke 99% growth inhibition on 4^th^ instar *S. exigua* larvae. Our results showed that none of these genes were found regulated in response to the lowest concentration of Vip3Ca used. Conversely, when *S. exigua* larvae were exposed at 1000 or 10000 ng/cm^2^ of Vip3Ca, the number of regulated AMPs genes was 8 and 15, respectively. Our data agree with previous data obtained from Crava *et al*.^56^ in that the response of the *S. exigua* larvae might be associated to the level of cell damage produced by the different *B. thuringiensis* proteins used and not to the mode of action of these proteins.

Genes from the three main immune signaling pathways (*Toll, Imd*, and *JAK-STAT*) were also represented in our study. The results showed that neither the Toll-like receptor gene nor the *Imd* gene were found to be regulated at any of the 3 concentrations tested. In contrast, the gene encoding a component of the JAK-STAT pathway was down-regulated after 24 h exposure to 1000 ng/cm^2^ of Vip3Ca. Additionally, the expression of this gene was also found down-regulated when larvae were exposed at 10000 ng/cm^2^ of Vip3Ca for lower times (3 h, 6 h, and 12 h). The JAK-STAT pathway has been shown to be involved in the activation of the midgut renewal by the proliferation and differentiation of the stem cells^59,60,68^. Thus, we speculate that the negative regulation of this pathway might be affecting the renewal of the midgut cells that have been damaged by the action of the Vip3Ca protein. This negative regulation might be a host defense response for gut healing processes after entomopathogen exposure^39^. The lack of regulation of the Toll and Imd immune signaling pathways was also described when S*. exigua* larvae were exposed to Vip3Aa protein^31^. It is worth to note that the activation of most of the immune signaling pathways relies mainly on post-translational modifications (e.g. phosphorylation) and to a lesser extent on transcriptional regulations^40,69^.

The effect of Vip3Aa and Vip3Ca proteins on the midgut epithelial cells was measured by determining the APN activity into the luminal fluid. The results showed that both Vip3 proteins cause APN shedding into the luminal fluid in *S. exigua* larvae. The ability of some *B. thuringiensis* proteins and other pore forming toxins (PFTs) to cause shedding of cell surface proteins has already been reported^70–72^. In agreement with our results, Valaitis *et al*.^70^ found that exposure to Vip3Aa induced shedding of APN in *Lymantria dispar* larvae. However, they hypothesized that the APN shedding would not be involved in host defense to *B. thuringiensis* proteins, since the inhibition of the APN shedding by cyclic AMP did not affect their toxicity^70^. Here we observed a clear dose-response relationship between the growth inhibition produced by increasing concentrations of Vip3Ca and the APN activity in the lumen of the larvae. This result supports that the depletion of the APN is stronger when the damage produced to the epithelial cells is larger. Moreover, when comparing the APN shedding observed after exposure using a concentration of either Vip3Aa or Vip3Ca proteins which produces 99% growth inhibition (100 and 1000 ng/cm^2^, respectively), the results showed that the luminal APN activity is higher in those larvae exposed to Vip3Aa than in those exposed to Vip3Ca. This result suggests that the cell damage produced by the Vip3Aa protein might be larger than the one produced by the Vip3Ca protein. Moreover, this result is in agreement with the results obtained in the gene expression analysis, since the exposure to Vip3Aa seems to regulate the expression of more genes that the exposure to Vip3Ca, at the same dose, in *S. exigua* larvae.

The analysis of the *S. exigua* midguts exposed to sublethal concentrations of Vip3Aa and Vip3Ca proteins by TUNEL assays showed the presence of TUNEL-positive cells at different concentrations of each protein (Fig. 6). TUNEL-positive cells were clearly observed at the highest concentration (10000 ng/cm^2^) of the Vip3Ca protein. In contrast, TUNEL-positive cells were detected at the lowest and intermediate concentrations of Vip3Aa (1, 10, and 100 ng/cm^2^). No TUNEL-positive cells were observed at the highest concentration of Vip3Aa (1000 ng/cm^2^), probably due to the fact that, at this concentration, epithelial cells might be responding by other mechanisms and/or by other signal-transduction pathways to respond to such an attack^73,74^ or it might happen that most of the cell membrane was disrupted. The histopathological effects produced in larvae from *Mamestra brassicae* after exposure to either Vip3Aa or Vip3Ca has been described by Gomis-Cebolla *et al*. (2016)^18^. The results showed that feeding with both Vip3 proteins produced disruption of the midgut epithelium, though the damage caused by Vip3Aa protein was detected earlier, in agreement with its higher toxicity. Consistent with our data, Tanaka *et al*.^55^ reported the presence of TUNEL-positive midgut cells in *B. mori* larvae when treated with sublethal concentrations of Cry1Aa and few TUNEL-positive cells when larvae were exposed to lethal concentrations. On the basis of these observations, we can speculate that disruption of midgut cell membranes, by the pore formation activity, is a main event that occurs when the insect species are exposed to a high dose of an active *B. thuringiensis* protein. Nevertheless, other events, such as apoptosis, may happen when larvae are exposed to sublethal doses. Thus, apoptotic events might involve a host defense response to the damage produced by the toxic agent, leading to renewal of the epithelial layer^39,73^.

Although the mode of action of Vip3 proteins is still not completely resolved, it is commonly accepted that, similarly to Cry proteins, they bind to specific receptors and form pores in the brush border of the epithelial cells^13,15,17,18^. Additionally, some studies have reported that Cry proteins can activate different intracellular cascade pathways, leading to apoptotic cell death^10,51,52,54,55^. The ability to produce pores in their target cells and also to activate different intracellular cascade pathways has also been described for other toxins (e.g. aerolysin and alpha-toxin) produced by other bacteria^75,76^. Apoptosis is a special and highly regulated type of programmed cell death that can be induced by different factors. Apoptosis has a fundamental role in biological process such as: development, tissue homeostasis, DNA damage response, and immune response^77^. In mammalian cells, PFTs kill cells by two different mechanisms: (1) apoptosis, characterized by the activation of initiator caspases that trigger effector- caspases to cleave cellular substrates, and (2) inflammatory responses by the activation of inflammatory caspases^78^. Caspases (cysteine-dependent aspartate-specific proteases) are a family of evolutionary conserved proteins that have been described for playing a key role in apoptosis^79^. In addition to the TUNEL assays, apoptosis was monitored by the analysis of the expression levels of five *caspase* genes. The results showed that expression level of the *Se-Caspase-4* was highly up-regulated after 3 h, 6 h, and 12 h. This gene has special sequence features and its function has not been assessed yet^79^. Nevertheless, as this gene is regulated after 3 h of exposure we can hypothesized that it might play a role in the pro-apoptotic proteolytic cascade as an initiator caspase. The expression levels of the initiator *Se-Caspase-6* and the effector *Se-Caspases-1* and *-2* genes were found up-regulated after 12 h of Vip3Ca exposure. The expression levels of the gene coding *Se-Caspase-*3 was not regulated at the different times tested, indicating that maybe it would not be involved in the host response to Vip3 exposure.

Here we show for the first time that Vip3Aa and Vip3Ca trigger apoptosis in *S. exigua* midgut epithelial cells *in vivo*. However, further research will be required to define the apoptotic signal transduction pathway induced by both Vip3 proteins in *S. exigua* larvae. It was recently reported that Vip3Aa can induce apoptosis in Sf9 cultured cells and that this is mediated by the mitochondrial and caspase dependent pathways^53^. Portugal *et al*.^52^ showed that Cry1A proteins induced apoptosis in CF1 cultured cells. Interestingly, the authors observed that the pore formation activity of the Cry1A proteins is necessary to induce apoptosis. It is possible that, with Vip3 proteins, pore formation is also a necessary step for cells to enter the apoptotic pathway.

In summary, the results from the present study show that exposure of *S. exigua* larvae to sublethal concentrations of Vip3Ca (a protein with low activity against this insect) activates different insect response pathways which trigger the regulation of some genes (such as the antimicrobial effectors, *caspases*), induces APN shedding, and triggers other signals that lead to apoptotic cell death. Understanding the host response process to the *B. thuringiensis* proteins currently used in insect control will help to shed light on insect defensive mechanisms to toxic agents.

## Materials and Methods

### Insects

Larvae from the *S. exigua* FRA colony were kindly supplied by M. López-Ferber, INRA (St Christol les Alés, France). The insects were reared on artificial diet at 25 °C with a relative humidity of 70% RH and a photoperiod of 16 h/8 h (light/dark). The FRA colony had been maintained for more than 10 years without exposure to pathogens^80^.

### Expression and purification of Vip3Aa and Vip3Ca proteins

The Vip3Aa protein (NCBI accession No. AAW65132) was overexpressed in recombinant *Escherichia coli* BL21 carrying the *vip3Aa*16 gene. The Vip3Ca protein (NCBI accession No. AEE98106) was overexpressed in *E. coli* WK6 carrying the expression vector pMaab 10 (kindly supplied by Bayer CropScience N.V., Ghent, Belgium). Protein expression and lysis of Vip3Aa and Vip3Ca was carried out following the conditions described by Abdelkefi-Mesrati *et al*.^81^ and Gomis-Cebolla *et al*. (2016)^18^, respectively.

Vip3Aa and Vip3Ca proteins in the cell lysates were further purified by isoelectric point precipitation (Ipp) as described by Chakroun *et al*.^82^. The pH of the lysates was lowered with acetic acid to pH 5.5 for Vip3Aa, and to pH 5.9 for Vip3Ca. After centrifugation, the precipitated proteins were resuspended in solubilization buffer (20 mM Tris-HCl, 150 mM NaCl, pH 9) and dialyzed against the same buffer overnight. The amount of Vip3 proteins was quantified by densitometry after SDS-PAGE electrophoresis using the TotalLab 1D *v*13.01 software. As a control, the empty *E. coli* WK6 strain was cultured and processed in the same way as described above for the Vip3Ca producing strain.

### Growth inhibition assays

Susceptibility of newly molted 4^th^ instar *S. exigua* larvae to Vip3Ca was determined by growth inhibition assays using surface contamination method as previously described^80^. Sixteen larvae were individually exposed to each concentration for 24 h. As a control, 16 larvae were exposed to a protein preparation from the empty *E. coli* WK6 strain. For this, the pellet obtained after the Ipp of the empty *E. coli* WK6 was prepared in the same way as with the Vip3Ca protein. Five independent biological replicates were performed. The percentage of growth inhibition (% GI) was calculated as described Herrero *et al*.^83^, using the formula: % GI = [1 − (RG_t_/RG_c_)] × 100, where RG_t_ and RG_c_ represent the relative growth of larvae after exposure to either Vip3Ca or the empty *E. coli* WK6 strain preparation (control larvae), respectively. Relative growth was calculated as RG = [(W_1_ − W_0_)/W_0_], where W_0_ and W_1_ are the initial and final weight of the larva, respectively. The effective concentrations which produced a reduction in the larval growth of 50% and 99% were calculated using the GraphPad Prism *v*. 5.1 (GraphPad Inc., La Jolla, CA, US) using a non-linear logistic model (Hill equation).

### Effects on larva development after Vip3 protein exposure

The effect of Vip3Ca and Vip3Aa proteins on two parameters of fitness was determined: time to pupation and percentage of pupation. Sixteen 4^th^ instar larvae were individually exposed to 10, 100, 1000, and 10000 ng/cm^2^ of Vip3Ca or to 1, 10, 100, and 1000 ng/cm^2^ of Vip3Aa. As a control, 16 larvae were exposed to a protein preparation from the empty *E. coli* WK6 strain. After 24 h exposure, larvae were transferred to non-intoxicated diet to complete their development. Three independent biological replicates were performed. The developmental time from 4^th^ instar larvae to pupa and the number of pupae was daily recorded.

### Gene expression analysis

The expression profile of 47*S. exigua* genes, which were previously described as responding to a Vip3Aa challenge or other pathogens^31,56,57^ (Supplementary Table S1), was analyzed under two different conditions: WK6 exposed (used as a control) and Vip3Ca exposed. For this purpose, sixteen newly molted 4^th^ instar larvae were individually exposed to 100, 1000, and 10000 ng/cm^2^ of Vip3Ca for 24 h. For the analysis of the expression levels of apoptosis-related genes, five *S. exigua caspase* genes were selected^79^ (Supplementary Table S2). Six newly molted 4^th^ instar larvae were individually exposed to 10000 ng/cm^2^ of Vip3Ca for 3 h, 6 h, 12 h and 24 h. Then, larvae were dissected and midguts pooled and stored at −80 °C until used. Three independent biological replicates were performed.

RNA was purified from larval midguts using the RNAzol RT reagent (Sigma-Aldrich, St. Louis, MO, US) according to the manufacturer’s instructions. Purified RNA (0.5 μg) was then treated with DNase I (Invitrogen, Carlsbad, CA, US) and subsequently reverse-transcribed to cDNA using oligo-(dT) primers and the SuperScript II Reverse Transcriptase (Invitrogen), following the manufacturer’s protocol. Quantitative real-time PCR (qRT-PCR) was carried out using EvaGreen® (Biotis, Vilnius, Lithuania) following standard protocols and measured in an ABI Prism 7700 Sequence Detection System (Applied Biosystems). Each reaction was performed in a final volume of 20 μl, which contained 4 μl of cDNA (300 ng). Forward and reverse primers were added to a final concentration of 300 pM. Primers used in this study are described in Supplementary Tables S1 and S2. The 47 selected genes analyzed in this study, including the house-keeping gene, were previously designed and their efficiency tested by other authors^31,56,57^. Specific primers for the five *S. exigua caspase* genes used in this study were designed on the basis of their sequences from the NCBI database (HQ328953, HQ328958, HQ328966, HQ328975, and HQ328993) using Primer Express Software from Applied Biosystems (Carlsbad, CA, US) (Supplementary Table S2). Prior to quantifying differential expression among different treatments, the efficiency of each pair of primers was evaluated by performing 3-fold dilution series experiments. The specific amplification of transcripts was verified by dissociation curve analysis. The Rest MCS software (version 2) was used to obtain the expression ratios (-fold change)^84^.

### Measurement of aminopeptidase activity in the midgut lumen

The APN activity in the midgut lumen from *S. exigua* larva treated with either Vip3Ca or Vip3Aa proteins was measured as a marker to evaluate the damage produced by the proteins after 24 h exposure. At least three independent replicates were performed for each condition. In each replicate, sixteen 4^th^ instar newly molted larvae were exposed to four different concentrations of Vip3Ca (10 100, 1000, and 10000 ng/cm^2^) and to one single concentration of Vip3Aa (100 ng/cm^2^). The concentration of Vip3Aa used produced a 99% of growth inhibition according to a previous study^31^. As a control, WK6 proteins prepared as for the Vip3Ca sample were used. The contents from at least 10 midguts (for each condition) were obtained and transferred into 100 μl of 50 mM Tris-HCl, pH 7.5, 1 mM PMSF. Then, midgut contents were vortexed for 30 s, centrifuged at 21 000 x *g* for 5 min at 4 °C, and the supernatant was used for activity assays. Protein concentration was determined by Bradford^85^. APN activity was determined using 4 mM L-leucyl-p-nitroanilide in 50 mM Tris-HCl (pH 7.5) buffer as substrate. The released of p-nitroanilide was monitored at 405 nm for 2 min using a Tecan Infinite 200 plate reader (Switzerland). An extinction coefficient of 9.9 mM^−1^ cm^−1^ for p-nitroanilide was used.

### Sectioning of insect tissues and TUNEL staining

Fragmentation of the DNA in the midgut epithelial cells from *S. exigua* larva exposed to either Vip3Ca or Vip3Aa proteins was measured as a marker of apoptosis. DNA fragmentation was measured using the principle of TUNEL (TdT-mediated dUTP Nick-End Labeling), which consists on the catalytical incorporation of a labelled dUTP (2′-deoxyuridine 5′-triphosphate) at the 3′- hydroxyl (-OH) group of the DNA end using a terminal deoxynucleotidyl transferase. For TUNEL staining assays, 4^th^ instar newly molted larvae were treated for 24 h under four different conditions: WK6 exposed (used as a control), Vip3Ca exposed, Vip3Aa exposed, and starving (larvae kept without food for 24 h). In each assay, 16 larvae were exposed to four different concentrations of Vip3Ca (10, 100, 1000, and 10000 ng/cm^2^) or Vip3Aa (1, 10, 100, and 1000 ng/cm^2^). Three independent biological replicates for each treatment were performed. After 24 h exposure, only larvae actively eating (as determined by observing the food bites) were selected, flash frozen and kept at −80 °C until used. Sections of 10 μm were prepared by the microscopy facilities at the Universitat de València using the cryostat microtome Leica CM 1510 S. Slides with the tissue sections were stored at −20 °C until used. Tissue sections were treated with the DeadEnd^TM^ Fluorimentric TUNEL system (Promega) following the manufacturer’s instructions. Nuclear DNA was stained with DAPI (4′,6-diamidino-2-phenylindole) as described by Chazotte^86^. Coverslips were mounted using mounting medium from Sigma. Tissue sections were then examined using Leica DMI2500 microscope equipped with a digital color camera (Leica DFC300 FX). Tissue sections were stained with hematoxylin and eosin as was described elsewhere^87^ to check the quality of the midgut sections (Supplementary Fig. S3).

## Electronic supplementary material

Supplementary information 
