# Supplementary material for: Changes in gene expression and apoptotic response in Spodoptera exigua larvae exposed to sublethal concentrations of Vip3 insecticidal proteins
Source: Sci Rep. 2017 Nov 24;7:16245. doi: 10.1038/s41598-017-16406-1 (PMC5701239; doi:10.1038/s41598-017-16406-1)
Supplement: Supplementary file 1 — Supplementary information [file 41598_2017_16406_MOESM1_ESM.pdf]

Supplementary material

Changes in gene expression and apoptotic response in *Spodoptera exigua* larvae exposed to sublethal concentrations of Vip3 insecticidal proteins

Patricia Hernández-Martínez, Joaquín Gomis-Cebolla, Juan Ferré and Baltasar Escriche*

ERI de Biotecnología y Biomedicina (BIOTECMED), Department of Genetics, Universitat de València. Dr Moliner, 50, Burjassot (46100), Spain.

Supplementary Information:

Supplementary Figure 1. Venn diagram showing up-regulated genes (panel a) and down-regulated genes (panel b) after 24 h challenged at 100, 1000, and 10000 ng/cm^2 of Vip3Ca.

Supplementary Figure 2. Correlation analysis between larval growth inhibition and the APN activity in the luminal fluid after exposure to four different concentrations of Vip3Ca protein for 24 h. Pearson r and p-value are shown in the graph.

Supplementary Figure 3. Midgut tissue sections of *S. exigua* exposed for 24 h to Vip3Aa and Vip3Ca proteins were stained with hematoxylin and eosin. As controls, larvae fed with the empty vector (WK6) were used. Magnification was 100×. BM, basal membrane; AM, apical membrane and L, lumen.

Supplementary Table S1. List of primers for RT-qPCR used in this study and gene expression of each transcript after Vip3Ca challenged at 100, 1000, and 10000 ng/cm^2, respectively.

Supplementary Table S2. List of primers designed for the analysis of the expression levels of five apoptosis-related genes.

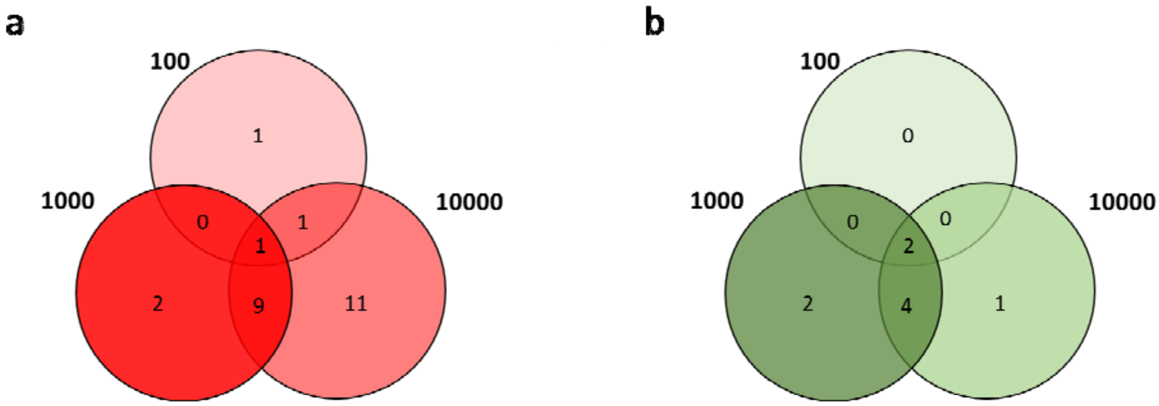

Supplementary Figure 1.

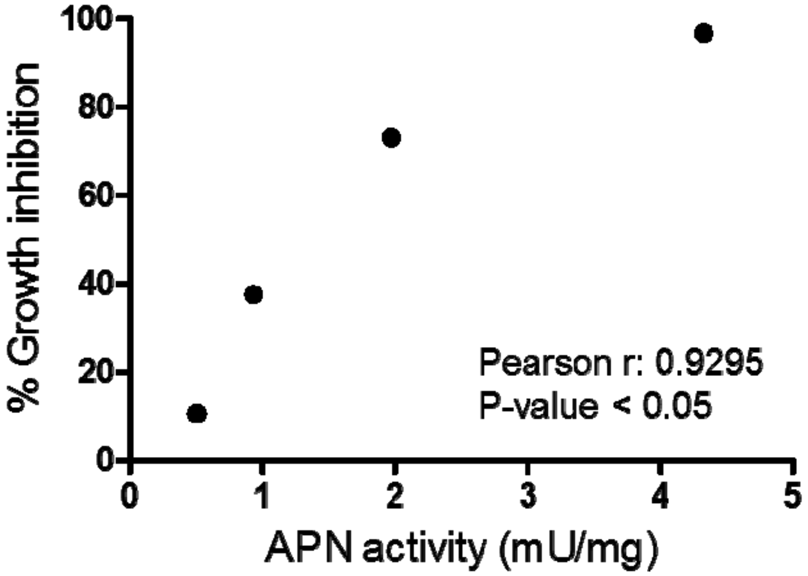

Supplementary Figure 2.

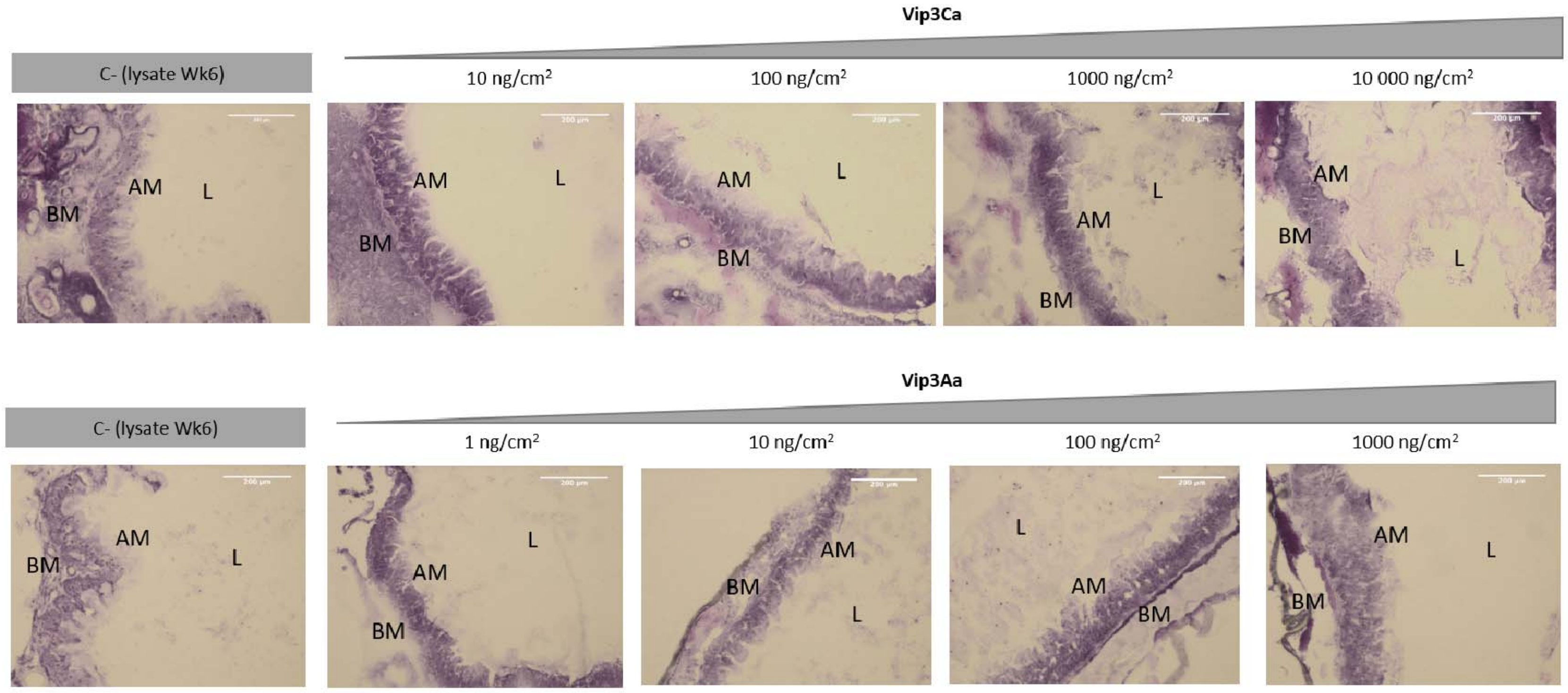

Supplementary Figure 3.

Supplementary Table S1: List of primers for RT-qPCR used in this study and gene expression of each transcript after Vip3Ca challenged at 100, 1000, and 10000 ng/cm^2, respectively.

Number of pair of primer	Target gene	Primers		Source	Fold change ± SD		
		Forward (5' → 3')	Reverse (5' → 3')		expose 100 ng/cm^2	expose 1000 ng/cm^2	expose 10 000 ng/cm^2
1	*ATP synthase*	GTTGCTGGTCTGGTGGGATT	AGGCCTCAGACACCATTGAAA	Herrero et al., 2007	-	-	-
2	*Beta-glucan recognition protein*	AATTGGAAGCCATCTATCCTAAAGG	TGAGGTTTCCGTGGAATGC	Jakubowska et al., 2013	0.8 ± 0.6	5.0 ± 3.9*	2.7 ± 2.8
3	*Peptidoglycan recogniztion protein*	GTAGTACCGGAGTGTGTTAGTGATGAG	TTGTCCTATATCAGTGAATCCACGTT	Jakubowska et al., 2013	1.1 ± 0.8	1.7 ± 1.7	3.5 ± 4.4
4	*Prophenoloxidase activating enzyme*	AGCTGTGCGGCCCAGAT	TCGACACCGCAACATTCACT	Jakubowska et al., 2013	1.1 ± 0.7	3.0 ± 2.6	3.5 ± 3.6
5	*G-protein receptor*	GGCCGTCAGTGTGAAGAATATTAAGT	ACGGGAACAGCAAATTGTTGT	Jakubowska et al., 2013	1.3 ± 0.6	0.8 ± 0.9	1.0 ± 0.8
6	*TIN-ag-RP*	CGATGACTGTTGCCCAGACTAC	TGCAGCCCATGGTGTTATATTC	Jakubowska et al., 2013	0.8 ± 0.4	1.9 ± 1.7	2.6 ± 1.7
7	*Toll receptor*	TTCTTTAGTCTTTTCCAGAACATTGG	ACCTGATGCTGACAAAGACCTACA	Jakubowska et al., 2013	1.3 ± 0.7	0.3 ± 0.4	0.8 ± 0.5
8	*Imd*	GCTCCAAGGCCATCTACAGAGA	TCCTGATCTTCATTTTGATCTTGATT	Jakubowska et al., 2013	1.1 ± 1.1	0.06 ± 0.2	0.5 ± 0.3
9	*JAK-STAT*	CGCCCTTACAGGATCATCTCA	AGGCCGGATTCTAGGAGCTT	Jakubowska et al., 2013	0.7 ± 0.4	0.4 ± 0.2 *	0.5 ± 0.3
10	*SE_U12696 (REVIP)*	GGTCCAATTCCAACATGCACT	TGTAGGTCTTGTGAACGTGGTGT	Bel et al., 2013	0.1 ± 0.1 *	0.0015 ± 0.0014 *	0.006 ± 0.0033 *
11	*SE_U12832*	ACTGGTGCAGTTCCGAGCAT	AGCCCCAATACTGTGTCCCA	Bel et al., 2013	0.4 ± 0.3 *	0.07 ± 0.04 *	0.09 ± 0.06 *
12	*SE_U59986*	GCCATTGCCTTACCTTCTGG	GCTTCCAACAAAGTTCTCGTTGA	Bel et al., 2013	0.7 ± 0.4	0.07 ± 0.05 *	0.06 ± 0.03 *
13	*SE_U10224*	CGAAGGGAATGTTTGCGAAG	AGTTCGCTGACCAGAGAGTGC	Bel et al., 2013	0.6 ± 0.4	0.08 ± 0.04 *	0.2 ± 0.1 *
14	*SE_U08180*	ATTCGCCCGACCTCTTCAAT	TGTTTAGGATGAACTGGAACCATAAC	Bel et al., 2013	0.6 ± 0.4	0.06 ± 0.04 *	0.2 ± 0.1 *
15	*SE_U08346*	AGGTCATCTCCAGCTACGACG	CGTTGCACGATTCAAATTCG	Bel et al., 2013	0.6 ± 0.4	0.06 ± 0.04 *	0.1 ± 0.07 *
16	*SE_U56776*			Bel et al., 2013	5.9 ± 3.6 *	11.0 ± 36.0	29.3 ± 34.6 *
17	*SE_U20473*	CGGCCAAGAATTAGTTTCCAAA	AGACCGGGTACTCTGGCGTA	Bel et al., 2013	9.3 ± 6.9	6.4 ± 3.5 *	6.9 ± 6.1 *
18	*SE_U33476*	CAGTACAATGGCCGCTCTCAA	AAGGCAATGAGGAGCAGCAC	Bel et al., 2013	0.8 ± 0.9	9.8 ± 12.9	19.3 ± 11.6 *
19	*SE_17986*	CGAGTGCACCATGAACACCT	ATGACGGCGAGGAAAGAGAG	Bel et al., 2013	1.8 ± 1.3	19.2 ± 11.6 *	10.4 ± 8.9 *
20	*SE_U08322*	GCCGCTAAGAATGCAGCTAAA	TGATGCCCGTGGAAGCTT	Bel et al., 2013	8.8 ± 5.9 *	11.3 ± 5.6 *	6.7 ± 5.7 *
21	*SE_U08997*	CTCCCCGAAGCTGAGACCT	TGGTCTCCGGCTTTATTGGA	Bel et al., 2013	1.4 ± 0.4	1.4 ± 0.6	2.3 ± 1.2 *
22	*SE_13239*	TTGGGCATCAAGTCGCTAGA	GTCCCCCTTGATCTCGTCAA	Bel et al., 2013	2.9 ± 2.3	20.9 ± 23.0 *	7.8 ± 5.6 *
23	*SE_U09334*	CAGTCGCCGGCCAAATAC	CGGGCTCGGCTTTATAGACC	Bel et al., 2013	5.2 ± 3.6 *	3.2 ± 3.6	1.8 ± 1.6
24	*SE_U06544*	TCAATTTCCAATAAAGCCGGA	TCTCGTGCTCAGCAATGTGC	Bel et al., 2013	1.1 ± 1.0	1.2 ± 1.1	1.1 ± 1.0
25	*Gloverin*	CGAGGTGGCTACAAACAAGAC	CATATGCCTGGCCTTGAAG	Crava et al., 2015	1.1 ± 1.0	4.2 ± 3.6	31.7 ± 5.0 *
26	*Attacin 1*	CGTTCTTAGACCGCAAGGAC	CACGGAAGTGGTCGGGCT	Crava et al., 2015	1.5 ± 2.3	1.6 ± 2.6	2.2 ± 2.0
27	*Attacin 3*	CGGTTTATCAGCACCATTCGGT	CGCCTGGCAGCATCAAAG	Crava et al., 2015	1.3 ± 1.6	0.5 ± 0.7	2.9 ± 2.6
28	*Lebocin 1*	CACTACACCTGCCTGACTACA	GGCGAGGGTTGAAGGGA	Crava et al., 2015	1.2 ± 1.6	35.6 ± 19.5 *	14.7 ± 10.5 *
29	*Cecropin A1*	GTCATCGTAATCATCACATCAACTAC	ACGGCAGGCAGTTGCTCAG	Crava et al., 2015	0.2 ± 0.4	1.9 ± 2.1	1.2 ± 1.1
30	*Cecropin B*	GGATAAGCTGGTCTCCAAACAC	GTGTGCCAACTTATTCGAGAAC	Crava et al., 2015	1.3 ± 1.1	5.2 ± 8.9	2.5 ± 2.3 *
31	*Cecropin C*	CAGTGAGGAAGACTAGACGGC	ATGGAGCGTATACAAATGAACG	Crava et al., 2015	1.1 ± 0.9	4.5 ± 7.0	3,05 ± 2.8 *
32	*Cecropin D*	GCCAAAGCGCTAGGAAAGTAG	TCTGTTGCTGACTATTGAAGTAGG	Crava et al., 2015	0.6 ± 0.5	3.2 ± 5.3	2.7 ± 2.3
33	*Cecropin E*	TGGCCGTTGTGGGATCAG	GTATGTGTCAGGTCATAGGGACT	Crava et al., 2015	1.3 ± 1.1	1.2 ± 1.9	8,1 ± 8.3 *
34	*Cecropin F*	CCAAGGCGCTAGGATAAAC	GGCGGAATGAGTATTATGAGGT	Crava et al., 2015	1.1 ± 0.8	0.9 ± 3.2	-2.2 ± 0.2
35	*Spodoptericn*	TCGTGCGATTTCGAAGAAGC	GCAGATGCCGTAAGTGTAACCT	Crava et al., 2015	3.4 ± 3.7	10.2 ± 4.6 *	21,9 ± 28.4 *
36	*LYZ 1*	GAATCATGCAAAAGCTAACGGT	TGCCTCGCAATGCAAGCA	Crava et al., 2015	0.8 ± 0.7	3.0 ± 4.9	2.2 ± 1.9 *
37	*LYZ 2*	GACGAATTGCGATTTAGTTCAC	GAGCACTCTCACTGTTTACCAGAC	Crava et al., 2015	1.2 ± 0.9	3.7 ± 6.5	2.8 ± 2.1 *
38	*LYZ 3*	CCTAATTGAAGCGGAGGGTT	GTGGGAACCGTCTGAATTCG	Crava et al., 2015	1.4 ± 1.2	2.4 ± 4.7	11.1 ± 7.3 *
39	*LLP 1*	GCCTGATATTGAGAAATGTCCA	CTTGCCGTTCTCTTGTCTCTAGG	Crava et al., 2015	0.5 ± 0.4	0.5 ± 0.4	0.5 ± 0.3 *
40	*LLP 2*	CGCGGTCCAGCACTAAGAC	CGCCTAGATCTTCTCAACCTGG	Crava et al., 2015	1.1 ± 0.5	0.8 ± 0.7	-2.0 ± 0.5
41	*Diapausin A1*	GCGGTAGAATGGACTGTTACTGATG	CAAGAAGGTTATCACGAATACG	Crava et al., 2015	1.8 ± 1.4	2.8 ± 2.8*	21.6 ± 15.3 *
42	*Diapausin A2*	GCGGTAGAATGGACTGTTACTGATG	GCAAGTCAGGAATACTAAAGGGC	Crava et al., 2015	1.1 ± 0.6	0.6 ± 0.6	4.6 ± 4.0 *
43	*Diapausin A3*	GCGGTAGAATGGACTGTTACTGATG	CTAGAGAGCTGCGTTGTTCAC	Crava et al., 2015	2.2 ± 1.3	0.8 ± 0.7	29.0 ± 16.3 *
44	*Diapausin A6*	GCGGTAGAATGGACTGTTACTGATG	ATTAGGTTTCTAGGCTTGTGTCAC	Crava et al., 2015	3.1 ± 3.2	39.5 ± 49.0*	46.4 ± 38.8*
45	*Diapausin A7*	GCGGTAGAATGGACTGTTACTGATG	TAAGAAGATCCTCCACTACAAGG	Crava et al., 2015	3.9 ± 3.4	21.8 ± 16.4*	46.5 ± 40.8*
46	*Cobatoxin 1*	TCGAGGAGGTGGGAGATGTC	CGAACGGCTGGAGACTCTTC	Crava et al., 2015	0.8 ± 0.6	5.3 ± 3.4 *	4.7 ± 6.9
47	*Cobatoxin 2*	GAAGCTCGTATTGTTTGTCGT	CCTCAGCAAGTCGTCAATG	Crava et al., 2015	0.4 ± 0.2	0.4 ± 0.2*	0.8 ± 0.8
48	*Moricin*	AAGCGGCTCCAGGAAAGATACC	ATTGCCCGGAGACCTTTACCAA	Crava et al., 2015	1.6 ± 1.6	4.3 ± 2.8*	6.2 ± 6.6 *

* Genes whose expression was significantly up-(box in red) or down-regulated (box in green) after Vip3Ca challenge

Supplementary Table S2: List of primers design for the analysis of the expression levels of five apoptosis-related genes.

Target gene	Name	Length	Tm	%GC	5'-3' sequence	Amp Length
Se-Caspase-1	Se-Caspase-1 F	20	62	60	GCTTGAAGTCGCGTACTGGC	
	Se-Caspase-1 R	19	61	63	GTCTGCAGTCTGCTGGACG	137
Se-Caspase-2	Se-Caspase-2 F	20	59	55	ATCGATATCCCACCACGAGC	
	Se-Caspase-2 R	21	58	48	CAACACTAAATCCCAACGCTG	82
Se-Caspase-3	Se-Caspase-3 F	23	64	57	CACATGCTGACTTCCTCGTGCTG	
	Se-Caspase-3 R	25	63	52	CAGGTCCTCATGATGTTCCTCCA	122
Se-Caspase-4	Se-Caspase-4 F	22	60	55	CGAGGTACGAAGATCACCCAAG	
	Se-Caspase-4 R	21	60	57	GAGATCAGACTCCACTGGCAG	111
Se-Caspase-6	Se-Caspase-6 F	20	59	55	GAAGCTCTCCTTACCTGCCA	
	Se-Caspase-6 R	20	59	50	AGTACTTGCGTGCTGCATTG	76
